# The Internal Secretion of the Ovary

**Published:** 1900-10-20

**Authors:** 


### THE INTERNAL SECRETION OF THE OVARY.

It has become an article of faith with many that the ovary, besides performing its ordinary and proper work of making eggs, also produces an internal secretion which is essential to what may be termed full womanhood. On this hypothesis are based a series of operative methods which aim at leaving bits of ovary or bits of tube in the hope of avoiding the evils of an artificial menopause. Dr. Arthur Johnstone,[1] of Cincinnati, Ohio, energetically controverts this idea. He holds that there is not an iota of proof that the ovary has any other function than the manufacture of eggs; that it is in no sense a gland like the thyroid or the thymus; that the ovary is active during intra-uterine life, Graafian follicles beginning to ripen at the sixth month of gestation; that during infancy they ripen occasionally and persist in the same ratio up to puberty; that during the child-bearing period the follicles ripen more rapidly, at least five or six a year, but at no time amount to as many as the number of menstruations in the year; and that during and after the menopause the Graafian follicles still continue to ripen; and he asks, "If it is a lack of an internal secretion that causes the nervous disturbances of the menopause, why is it that the little girl does not have them? He then goes on to state that if a woman's menstruation is delayed for any reason except pregnancy, she is very apt to have symptoms closely allied to those of change of life; and he declares that the internal secretion of the ovary is a myth. "It is the last stand of those who have believed that the ovary dominates everything." He thinks, therefore, that the ovary will have to be relegated to the function of producing eggs alone. "When we see every other organ in the body controlled by the central nervous system, why is it," he asks, "that we will persist in saying that this one little epithelial body contains all the functions known to womanhood?" Coming to the practical bearing of the matter upon operative work, he says, "Where an ovary and its accompanying tube can be left in a healthy condition, so that their functions can be easily and safely carried out, they should be preserved; but I have nothing but condemnation for those dangerous experiments which result in the leaving of a scrap of one ovary in one part of the abdomen and a piece of tube in another, or the transplantation of an ovary from one patient to another. Suppose that we could accomplish what we desire—the preservation of menstrual life — what good would it do? It is only postponing the menopause; the troubles that a woman has at the menopause are hereditary, and what difference does it make whether she goes through them early or late?" In conclusion, he says that all this furor about the internal secretion of the ovary only results in bad work, and gives a cloak for leaving patients in dangerous conditions. This is one side of the question. Needless to say the experience of many operators suggests very different conclusions.

[1] New York "Medical News," October 6th.

